# PDX Finder: A portal for patient-derived tumor xenograft model discovery

**DOI:** 10.1093/nar/gky984

**Published:** 2018-12-07

**Authors:** Nathalie Conte, Jeremy C Mason, Csaba Halmagyi, Steven Neuhauser, Abayomi Mosaku, Galabina Yordanova, Aikaterini Chatzipli, Dale A Begley, Debra M Krupke, Helen Parkinson, Terrence F Meehan, Carol C Bult

**Affiliations:** 1 European Molecular Biology Laboratory- European Bioinformatics Institute, Wellcome Trust Genome Campus, Hinxton, Cambridge CB10 1SD, UK; 2 The Jackson Laboratory, 600 Main Street, Bar Harbor, ME 04609, USA; 3 Wellcome Sanger Institute, Wellcome Genome Campus, Hinxton, Cambridge CB10 1SA, UK

## Abstract

Patient-derived tumor xenograft (PDX) mouse models are a versatile oncology research platform for studying tumor biology and for testing chemotherapeutic approaches tailored to genomic characteristics of individual patients’ tumors. PDX models are generated and distributed by a diverse group of academic labs, multi-institution consortia and contract research organizations. The distributed nature of PDX repositories and the use of different metadata standards for describing model characteristics presents a significant challenge to identifying PDX models relevant to specific cancer research questions. The Jackson Laboratory and EMBL-EBI are addressing these challenges by co-developing PDX Finder, a comprehensive open global catalog of PDX models and their associated datasets. Within PDX Finder, model attributes are harmonized and integrated using a previously developed community minimal information standard to support consistent searching across the originating resources. Links to repositories are provided from the PDX Finder search results to facilitate model acquisition and/or collaboration. The PDX Finder resource currently contains information for 1985 PDX models of diverse cancers including those from large resources such as the Patient-Derived Models Repository, PDXNet and EurOPDX. Individuals or organizations that generate and distribute PDXs are invited to increase the ‘findability’ of their models by participating in the PDX Finder initiative at www.pdxfinder.org.

## INTRODUCTION

PDX models recapitulate many of the disease hallmarks of cancer patients and are increasingly being used to study therapeutics, tumor evolution and drug resistance mechanisms. PDX models are typically generated by the implantation of human tumor tissues or cells into severely immunodeficient mouse host strains. Tumors that engraft successfully are passaged further to generate cohorts of tumor-bearing mice for experimental studies. PDX models are generated and used by researchers in university, clinical and pharmaceutical industry settings as well as specialized commercial organizations. International consortia focusing on the use of PDX models including PDXNet and EurOPDX have recently been funded ensuring PDX models will continue to be an important contributor to understanding and treating cancer.

The distributed and diverse nature of PDX repositories as well as differences in associated metadata describing the models presents a significant challenge to researchers seeking to find PDXs that are relevant to specific cancer research questions. To address this issue, The Jackson Laboratory and the European Molecular Biological Laboratory-European Bioinformatics Institute (EMBL-EBI) have implemented PDX Finder (http://www.pdxfinder.org), a freely available and searchable catalog of global PDX repositories. The data model for PDX Finder is based on the minimal information standard for PDX models developed in collaboration with a broad range of stakeholders who create and/or use PDX models in basic and pre-clinical cancer research (1). PDX Finder currently provides access to information 1985 PDX models in 8 repositories around the world, including NCI’s Patient Derived Model Repository, The Jackson Laboratory's PDX Resource, members of the EurOPDX Consortium and members of NCI’s PDXNet. Here we describe the implementation of PDX Finder and illustrate how it can be used to locate relevant PDX models. We also provide information on how investigators with small or large repositories of PDX models can have their resource indexed in PDX Finder to improve the visibility of their models.

## PDX FINDER FEATURES AND FUNCTIONALITY

To ensure PDX Finder reflects the diverse needs and goals of researchers who use PDX models, we adopted a user-centred design process. User-centred design involved stakeholder and user interviews as well as workshops that allowed us, in the early stage of the project, to identify and characterize our main groups of users, suggest typical use cases and identify priority requirements for the users. The three priority features to emerge from the requirements gathering process were:

1. Availability of high level summaries (graphical and tabular) providing an overview of all the models and repositories included in PDX Finder.
2. Search forms that allow researchers to find PDX models based on diagnosis (e.g. colorectal cancer, invasive ductal carcinoma), cancer type (e.g. metastasis or primary), availability of specific datasets (e.g. mutation, dosing studies), molecular markers (e.g. KRAS V600E) and results from drug resistance/sensitivity dosing model studies (e.g. resistance to cetuximab).
3. Summary pages for individual models with details about how the model was generated, de-identified patient information, links to available data sets and model acquisition information.

### PDX Finder home page

The PDX Finder home page prominently features search capabilities allowing users to perform a lexical search over a number of categories and summary graphics of the resource. In the Find section, users can search for patient diagnoses to retrieve corresponding PDX models. In the Explore section, we also offer different points of entry to the models, represented by lists and bar charts that group models by ‘frequently mutated genes’, ‘drugs tested on PDX models’, ‘anatomical systems’ and individual providers. A ‘News and Events’ section on the bottom of page provides headline summaries with links to news items about the PDX resource as well as a Twitter feed where users can stay up to date with the latest developments to the resource. Finally, the top menu bar provides details about the PDX Finder project, including our information gathering process and the submit/contact section that contains instructions on how to submit PDX model data to the resource.

### Search functions

**Figure 1.**
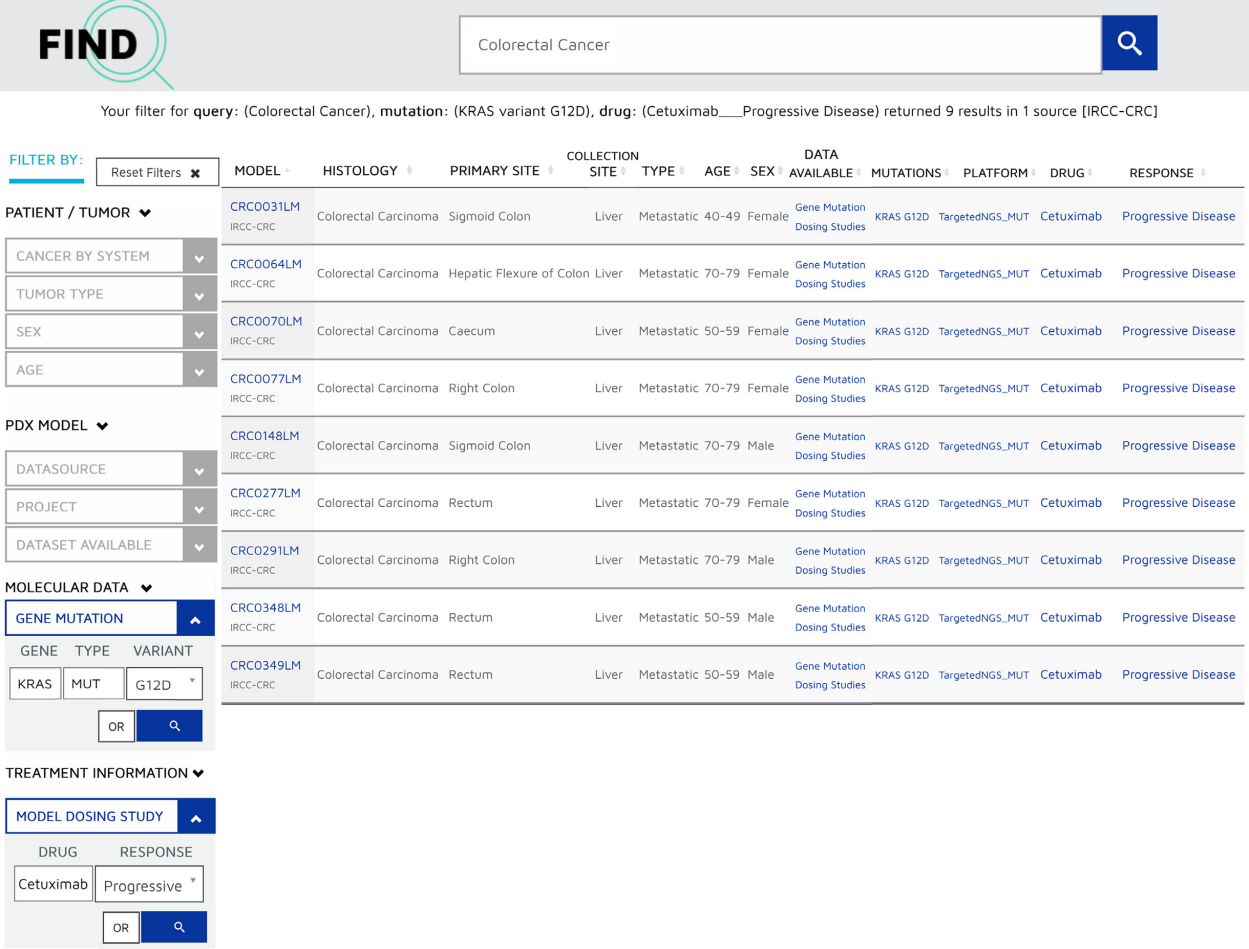
PDX Model Search page. Example of a search output corresponding to a query for colorectal cancer PDX model with KRAS G12D mutations and resistance to cetuximab.

Users navigate to the ‘Search Results’ page by searching for a diagnosis in the search box or choosing a category from one of the graphical visualizations. Results are presented in a tabular format with each row depicting a model (Figure 1). Key features of the models are presented in columns and include model ID and provider, tumor diagnosis, critical patient tumor clinical data and links to available datasets. Multiple filters provided on the left margin of the page allow users to further specify the models they would like to appear in the results table. Filters are grouped within categories according to PDX MI standard (1) and can be selected by expanding a facet and further selecting one or more filters in the relevant sub-categories. The search functionality allows users to find the right model for their project and filter accordingly with their own specific criteria. For example, ‘find all colorectal cancer models with KRAS G12D mutation,’ will retrieve 68 models from two sources. Additional filtering using drug dosing sensitivity or resistance will further refine the search to make it more specific. ‘Find all colorectal cancer models with KRAS G12D mutations and resistance to cetuximab’ will retrieve nine models from one source (Figure 1). Additional filters allow selection of models linked to associated dataset types (genomic data, drug dosing or patient treatment) or as derived from a specific project (EurOPDX or PDXnet). For searches that return multiple models, the results can be exported in a tabular format. Users can navigate to a model page or to the data of interest by clicking on the unique PDX Model ID or data links in each row.

### Model, patient and data detail pages

**Figure 2.**
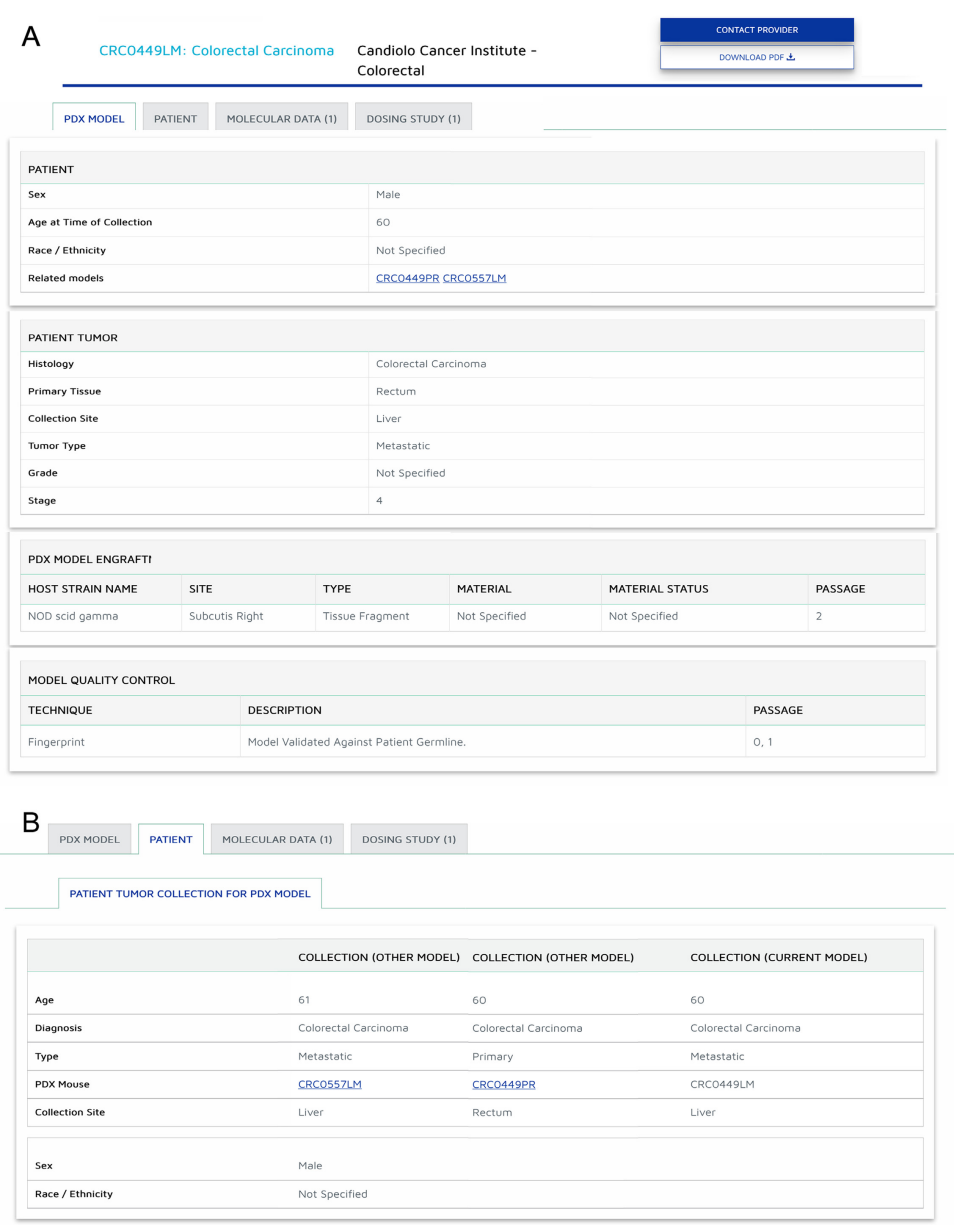
PDX Model and Patient Detail Pages. (**A**) Example of a PDX model detail page. A tabulated section beneath the overview provides summary views of clinical, model and validation information. Contact links at the top right allow users to directly contact the PDX Producer for further collaboration. (**B**) Example of a Patient Detail page that contains key clinical attributes about the tumor collection event(s) used to generate PDX models.

The PDX Model Detail page (Figure 2A) presents key features about the model. The top of the page displays the model ID and tumor histological classification as well as prominent links to the originating resource where users can find more information and contact the relevant institution for further collaboration. A tabulated section beneath the overview provides summary views of the models with clinical, model and validation information as well as views of additional data that may have been submitted with the PDX model including gene variant data or results from drug dosing studies.

The patient page (Figure 2B) displays information about the patient and the tumor collection event(s) used in generation of the PDX model. Key clinical tumor characteristics such as diagnosis, type (primary and metastatic), age and collection site are presented here. When patients are sampled at different times during their disease progression, clickable links to PDX models allow users to easily browse between these patient related models. Furthermore, details about treatment the patient received are included when that information is provided.

**Figure 3.**
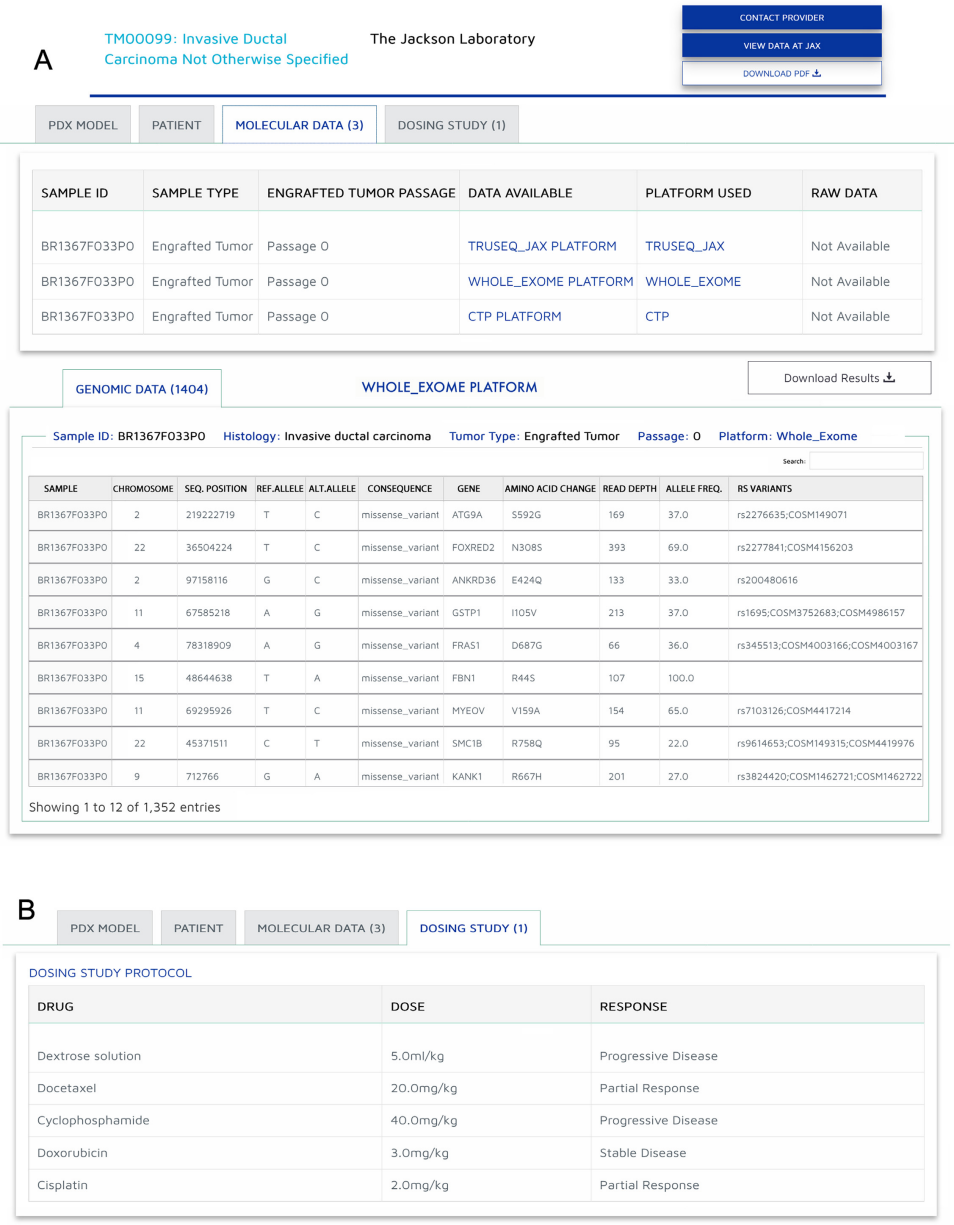
PDX Data pages. (**A**) The Molecular data page contains genomic data analysis files that where provided by the resources. A table summarises the types of data and files accessible, with links to the datasets. The results can be exported in a tabular format using the ‘Download Results’ icon. (**B**) The Drug dosing page summarise the drug-response results generated for a particular model. Links to protocols and platforms description are provided for all datasets to allow the user to understand how the data was generated and analysed.

The molecular data page contains genomic data analysis files that where provided by the resources. A table summarises the types of data and files accessible through the PDX Finder with links to external resources when available. Users can select a dataset to obtain a detailed view and can search their gene of interest using available search boxes (Figure 3A). Links to protocols and platforms description are provided to allow the user to understand how the data was generated and analysed.

The drug dosing page summarises the drug–response results generated for a particular model and provide links to the procedure description used to generate the dosing studies (Figure 3B).

## NOMENCLATURE AND METADATA STANDARDS

PDX Finder facilitates discovery of similar models across resources by the use and enforcement of nomenclature and metadata standards. Cancer type, diagnosis and other cancer attributes are represented by the NCI Thesaurus, a terminology resource that is maintained by the NCI (2). Names and symbols for human genes follow the nomenclature standards approved by the HUGO Gene Nomenclature Committee (3). Host mouse strain nomenclature follows the official guidelines established by the International Committee on Standardized Genetic Nomenclature for Mice (4). Drug/compound names use standards from NCIT(2), CHEBI (5), CHEMBL (6) and PubChem (7). In addition, we provide feedback to ontology developers to improve these resources for representing the complexities of PDX models.

## DATABASE AND SOFTWARE INFRASTRUCTURE

The PDX Finder web site was implemented using a combination of the WordPress content management system and a Java web application. The database for PDX Finder is a Neo4J graph database. To populate this database, bespoke Extraction, Transformation, Loading (ETL) software pipelines were written in Java to extract relevant attributes corresponding to the PDX minimal information standard from the data provided by the different PDX repositories. Software developed by the PDX Finder team is freely available under an Apache 2 license (https://www.apache.org/licenses/LICENSE-2.0) and the PDX Finder source code is available at GitHub (https://github.com/pdxfinder).

A number of key attributes specified in the minimal information standard rely on different vocabularies and ontologies. To address this challenging harmonization issue, PDX Finder employs a semi-automated integration approach using resources at EMBL-EBI including the ZOOMA annotation tool (8) that maps free text annotations to ontology terms based on curated repositories of annotation knowledge. Transformed metadata is reviewed and reported to the submitters for validation and approval. Once approved, the transformed metadata describing a model is then loaded into the PDX Finder database and exposed to the users through the web interface.

One challenge in metadata harmonization is the mapping of biologically identical histological concepts harboring different names provided by several sources using their own classification. To harmonize those concepts and to support consistent searching across the originating resources, we sometimes aggregate histological term and primary tissue attributes to achieve a more specific terminology. For example, ‘Adenosquamous’, ‘adenosquamous carcinoma’, ‘Ad and SC carcinoma’ all share the same primary tissue of origin ‘lung’ allowing mapping to the NCIT ontological term ‘Adenosquamous Lung Carcinoma’. Furthermore, ontological association allows aggregation of concepts based on meaningful groupings like cancer by anatomical system or cell morphology. This allows users to search for ‘Lung Cancer’ models across all subclasses of Lung Cancer models in a single query, without having to look for each subtype individually.

## FUTURE DIRECTIONS

PDX Finder is a freely available global catalog of PDX models and associated data available from independent, distributed repositories. Future development of PDX Finder will focus on three areas: addition of new PDX repositories, coordination with other informatics groups and implementation of user-requested functionality.

### Adding new PDX repositories

Our primary focus in the near term is to increase the number of PDX models represented in PDX Finder by contacting individuals and organizations that maintain PDX repositories. To support scalability of data submission and processing to PDX Finder, we have produced a data submission template, available on request, that contains several modules based on our previously published PDX MI standard and includes relevant examples to guide users. If imputing PDX data via the template is not practical, we also provide a metadata checklist that can be used to develop direct database exports (Supplementary Table S1). In addition to metadata for models, we strongly encourage producers to submit genomic datasets and information related to drug dosing studies associated to their models submission to enhance the information we make discoverable for end users through PDX Finder.

### Coordination with other informatics resources

To facilitate data sustainability, maximize use of existing data and avoid redundancy, the PDX Finder team has initiated coordinating activities with existing molecular archives to deposit data generated from PDX Models. The data loading process can broker the submission of data to established archives in collaboration with the data providers. Data which access require application to a Data Access Committee approval will be submitted to relevant secure molecular archives such as the European Genome-Phenome Archive (9) or dbGAP (10). Depending on the data type, non-patient identifiable data will be submitted to other archive resources including the Sequence Read Archive (11), the European Nucleotide Archive (12) for nucleotide sequencing information; Gene Expression Omnibus (13) or ArrayExpress (14) for gene expression data or European Variation Archive (https://www.ebi.ac.uk/eva/) for genetic variation information. PDX Finder will also coordinate the submission of sample metadata to BioSamples database (15), or BioSample archive (16), which provides unique identifiers that are used to link varying types of data derived from the same sample. Integration of the PDX Finder datasets linked to their harmonized data in molecular archives will ensure that meta-analysis type of studies combine biologically comparable datasets from multiple sources.

### New functionality

Other areas of development will be determined by the needs of the PDX research community. We will continue to assess user needs by surveys and testing of the portal. As the number of informatics resources using PDX models grows, the community is best served by sharing solutions to common challenges. The PDX Finder team is committed to contributing to a data science environment of collaborative development and reuse of informatics tools. In planning the implementation of new functionality and user interfaces, the PDX Finder team first evaluates software developed by other groups to address common needs. For example, we are coordinating with several resources funded by NCI’s Information Technology for Cancer Research (ITCR) program, including cBioPortal for cancer genomic data visualisation (17) and the CIViC Database for clinically relevant annotation of cancer variants (18). In addition to leveraging external bioinformatics resources to enhance functionality of PDX Finder, our team is providing critical software components to support the PDXNet and the EurOPDX data platforms.

Future versions of the PDX Finder will capture additional attributes and ‘omics’ datasets. Given the recent success of immune checkpoint inhibitors in the treatment of cancer, ‘humanized immune system’ PDX models have a key role in immuno-oncology drug validation, so their accurate representation will need to contain additional attributes such as HLA status of the tumor and the donor of human cells. We are monitoring advances with specialists in the field and PDX Finder will capture those models as standard approaches in their generation and use emerge.

## COMMUNITY OUTREACH AND USER SUPPORT

The PDX Finder user help desk is available via email and user outreach is supported through Twitter and the PDX Finder News and Event section on the homepage. Researchers interested in listing their PDX repository in PDX Finder should contact the team using the submission email provided below. A checklist of the data we collect is included in Supplementary Table S1. The PDX Finder team will work directly with data providers to ensure their PDX models are represented accurately.

- PDX data submission email: submissions@pdxfinder.org
- Twitter: https://twitter.com/PDXFinder

## CITING PDX FINDER

For a general citation of PDX Finder, researchers should cite this article. In addition, the following citation format is suggested when referring to specific data about PDX models obtained from the PDX Finder web site: PDX Finder (http://www.pdxfinder.org); data retrieved in September 2018.

## Supplementary Material

Supplementary Data
